# Safety of exercise for adults with thoracic aortic aneurysms and dissections

**DOI:** 10.3389/fspor.2022.888534

**Published:** 2022-08-22

**Authors:** Jesse Li, Alexandra Boyd, Michael Huang, Joshua Berookhim, Siddharth K. Prakash

**Affiliations:** ^1^McGovern Medical School, Houston, TX, United States; ^2^Division of Cardiovascular Medicine, Department of Internal Medicine, McGovern Medical School, Houston, TX, United States

**Keywords:** thoracic aortic aneurysms, thoracic aortic dissections, isometric exercise, dynamic exercise, Ambulatory Blood Pressure Monitors

## Abstract

**Background:**

Current guidelines for patients with thoracic aortic aneurysms or dissections (TAD) restrict vigorous exertion with the intention to prevent acute aortic dissections. However, a safe threshold for exercise intensity has not been established for TAD patients. In this study, we measured exertional changes in systolic and diastolic blood pressure during isometric and dynamic exercises in a cohort of TAD patients to determine safety of moderate intensity exercise.

**Methods:**

Thirty-one adults with TAD and 14 controls were recruited from UTHealth outpatient clinics. All participants completed an exercise protocol consisting of two circuits of five moderate intensity exercises: hand grips, leg raises, bicep curls, stationary cycling, and wall sits. Blood pressure values were recorded during exercise using Spacelabs OnTrak Ambulatory Blood Pressure monitors. Perceived exertion during each exercise was measured using the Borg CR-10 scale.

**Results:**

No significant differences in the maximum exertional systolic pressure, diastolic pressure, or change from baseline was found between the TAD and control groups. Higher amounts of self-reported weekly moderate activity level (MAL) in TAD correlated with lower exertional SBP during exercise. Higher Borg scores were associated with a greater change in systolic pressure.

**Conclusion:**

Moderate intensity exercise is safe and feasible for many TAD patients. Our data confirms that the Borg score may be a useful proxy for exercise intensity. In this study, we establish a reproducible exercise protocol that can be adapted to create individualized exercise regimens for TAD patients as part of a care plan to improve long-term cardiovascular health.

## Introduction

Thoracic aortic aneurysms and dissections (TAD) are a major cause of mortality in the general population with an estimated incidence of 3–4 cases per 100,000 persons per year (LeMaire and Russell, 2011). TAD is associated with congenital conditions such as bicuspid aortic valve, Marfan syndrome, Loeys-Dietz syndrome, and vascular Ehler-Danlos syndrome. Thirty-day mortality and long-term survival of TAD patients have improved due to advances in detection, medical therapy, and prophylactic aortic repair (Melvinsdottir et al., 2016). TAD survivors are often young people who intend to maintain an active lifestyle but encounter anxiety and uncertainty about exercise. Anecdotal reports about acute aortic dissections that occurred during vigorous exercises such as weightlifting may provoke this anxiety. TAD patients are frequently counseled to minimize strenuous activities due to their perceived risk for sudden death. However, sedentary behaviors predispose to atherosclerotic cardiovascular diseases and reduced quality of life (Pasadyn et al., 2021). Tight control of hypertension is a critical component of prophylaxis against aortic dissection and the need for reoperation in TAD patients (Melby et al., 2013). There is strong evidence that regular exercise lowers blood pressure and improves cardiovascular outcomes across a wide spectrum of cardiovascular diseases (Ghadieh and Saab, 2015). On a weekly basis, at least 150 min of moderate aerobic exercise and 20 min of isometric exercise can reduce systolic blood pressure (Owen et al., 2010; Chaddha et al., 2014). Daily moderate intensity exercise may even lead to a dose-dependent reduction in cardiovascular mortality (Paffenbarger et al., 1986). However, these potential benefits need to be balanced against the potential risks of exertional hypertension, which can trigger an acute dissection. There are no evidence-based exercise guidelines for patients with TAD that account for these competing concerns. Therefore, we determined the safety and feasibility of exercise in a cohort of TAD patients with extensive vascular disease. We selected five different isometric and dynamic maneuvers to replicate common exercises that patients frequently ask about (Chaddha et al., 2014). We also evaluated how blood pressure responses to exercise varied with habitual activity levels, medication use, and disease phenotype (Chrysant, 2010). We demonstrate how data from this reproducible protocol can be used to diagnose exertional hypertension and develop individualized exercise programs for TAD patients.

## Methods

Adult patients 18 years or older who were diagnosed with TAD or gene mutations that predispose to TAD were eligible for this study. Individuals were excluded if they were less than 18 years old or had baseline systolic blood pressure (SBP) greater than 160 mmHg, had a myocardial infarction, stroke, or aortic surgery within the previous year, or were unable to complete the study exercises due to physical limitations. At enrollment, research participants completed questionnaires that included: demographic data, self-reported medication usage, and a standard measure of weekly activities derived from the World Health Organization Global Physical Activity Questionnaire (Opoku-Acheampong et al., 2021). A subset of participants also completed one follow up survey after protocol completion about participation in exercise and perception of exercise safety. Additional clinical and imaging data were abstracted from medical records. All elements of the study protocol were reviewed and approved by the Committee for the Protection of Human Subjects at the University of Texas Health Science Center at Houston (UTHealth).

Study subjects were recruited from UTHealth outpatient clinics. All participants completed a standardized exercise protocol that included two circuits of five moderate intensity exercises: hand grips, leg raises, bicep curls, stationary cycling, and wall sits. Hand grip resistance level was determined as 40% of maximal exertion using the dominant hand. Leg raises were performed in a supine position with both heels elevated six inches above ground level. Bicep curls were performed with the dominant hand using 5 or 10 lb weights. Stationary cycling was performed at a target of 80–100 W. Wall sits were maintained with a ninety-degree angle between the back and lower legs. Perceived exertion during each exercise was measured using the Borg CR-10 scale (Borg, 1970), with a score of one representing minimal exertion and a score of ten indicating maximal exertion. Borg exertional scores were introduced after the onset of the study and obtained for twelve TAD patients. Cardiac rehabilitation facility staff and trained study personnel supervised all exercises. The protocol was promptly terminated if SBP increased to more than 230 mmHg, DBP increased to more than 120 mmHg, onset of chest pain, or at the request of the participant.

BP was measured three times at rest prior to the circuit, twice during each exercise, and once between each exercise. Measurements were manually triggered using OnTrak Ambulatory Blood Pressure Monitors (ABPM, Space Labs, Inc., Snoqualmie, WA) with the cuff on the non-dominant arm. Participants were sitting or lying down and engaged in steady state maneuvers throughout each measurement. Supervisors ensured that the measurement arm was immobilized while the cuff inflated as recommended in the AHA scientific statement on blood pressure measurement (Muntner et al., 2019). All exercises were initiated for 15 seconds prior to triggering the ABPM and maintained until the readings were completed. Data was analyzed using Sentinel software (v11, Space Labs, Inc., Snoqualmie, WA). Descriptive statistics were compared using chi-squared or Fisher's exact tests. Measurements were compared using two-tailed Student t-tests.

## Results

### Study cohort

This study enrolled 31 participants with TAD and 14 unaffected individuals without any history of TAD (Table 1). TAD cases consisted of thoracic aortic aneurysm (*n* = 18), thoracic aortic dissection (*n* = 8), elective open or endovascular repair of the thoracic aorta (*n* = 8), bicuspid aortic valve (*n* = 5), Turner syndrome (*n* = 4), Marfan syndrome (*n* = 2), and vascular Ehlers-Danlos syndrome (*n* = 2). Only three of 23 non-syndromic TAD cases had a known causative gene mutation (*FBN1, MYH11, TGFB2*). In all TAD cases, aortic imaging reports were reviewed to corroborate diagnoses and aortic phenotypes.

**Table 1:** Demographic characteristics of study participants.

	**TA Aneurysm** **(***n** =* 18)**	**TA Dissection** **(***n** =* 8)**	**TA Graft/repair** **(***n** =* 8)**	**BAV** **(***n** =* 5)**	**TS** **(***n** =* 4)**	**MFS** **(***n** =* 2)**	**vEDS** **(***n** =* 2)**	**Control** **(***n** =* 14)**
Age	51 (12)	46 (15)	47 (15)	38 (17)	27 (7)	40 (1)	40 (3)	35 (16)
Male/female	14/6	4/4	5/3	4/1	0/4	0/2	2/0	4/10
BMI	28.1 (8.2)	28.4 (8.3)	26.0 (3.9)	25.8 (2.0)	25.2 (0.7)	29.4 (17.5)	26.5 (6.1)	26.8 (3.7)
Weekly MAL	220 (298)	71 (137)	152 (176)	263 (252)	393 (210)	0 (0)	0 (0)	171 (186)
Weekly VAL	162 (187)	71 (111)	63 (109)	156 (197)	45 (77)	315 (403)	0 (0)	56 (84)
BB	8 (44%)	6 (75%)	5 (63%)	1 (20%)	0 (0%)	1 (50%)	1 (50%)	0 (0%)
Other AH	11 (61%)	5 (63%)	6 (75%)	1 (20%)	1 (33%)	0 (0%)	0 (0%)	0 (0%)

*Data shown as mean (SD). TA, Thoracic aortic; BAV, Bicuspid Aortic Valve; TS, Turner Syndrome; MFS, Marfan Syndrome; vEDS, vascular Ehlers-Danlos Syndrome. BMI, body mass index (kg/m^2^); Weekly MAL, weekly moderate activity level (min/week); weekly VAL, weekly vigorous activity level (min/week). Medication usage shown as the number of users (percentage of group) taking any dosage of listed medication category*.

### BP response in TAD cases compared to controls

**Figure 1:**
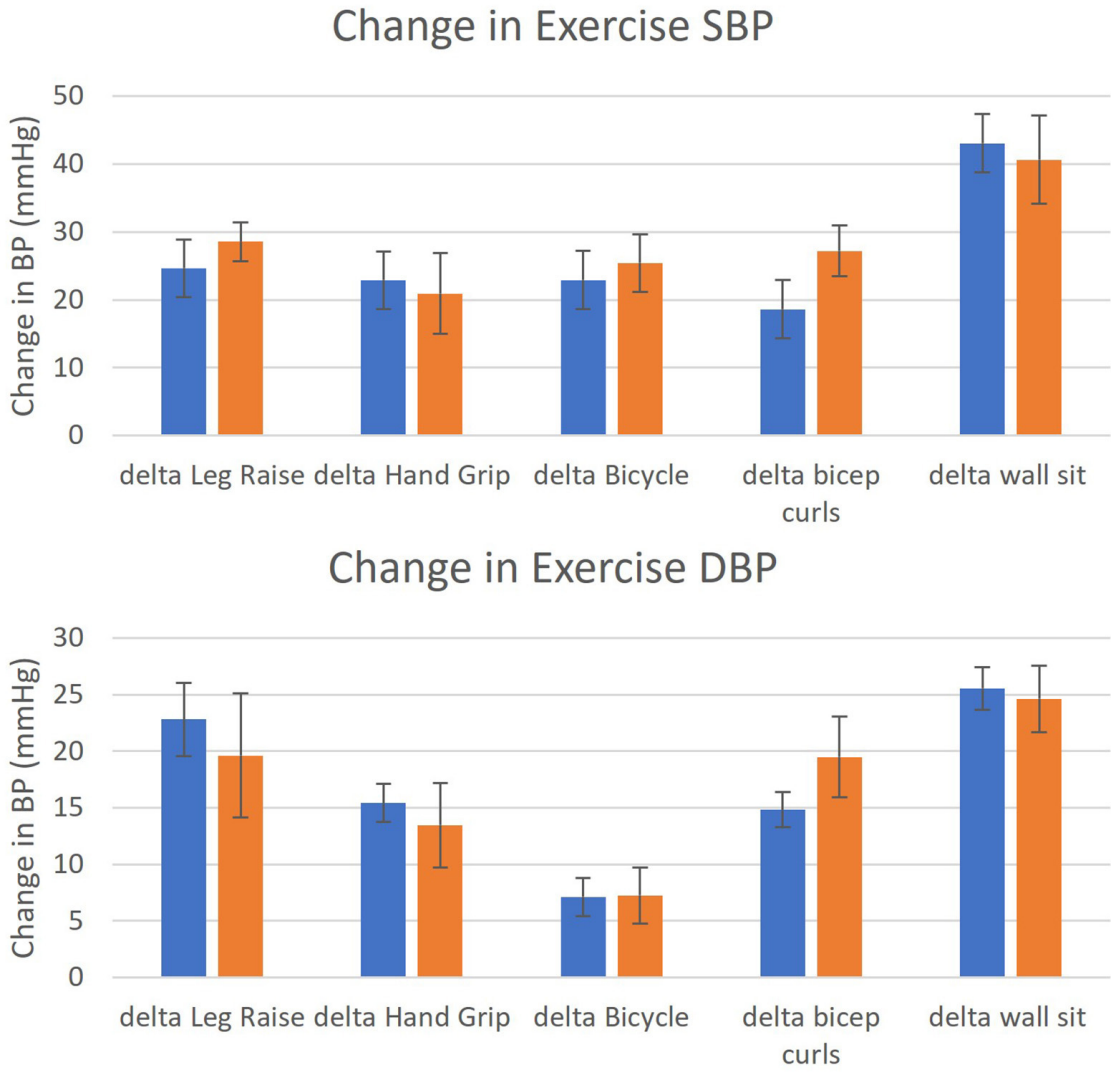
Mean change in exercise systolic blood pressure (SBP) and diastolic blood pressure (DBP) from baseline in TAD (*n* = 31) and controls (*n* = 14). Legend: TAD (blue); controls (orange). Y-axis: change in blood pressure during activity (mmHg); x-axis: as labeled, error bars: one standard error. Differences in SBP and DBP between TAD and controls were assessed with 2-tailed Student t tests with appropriate degrees of freedom.

There were no major differences found between the baseline SBP or DBP of cases (Mean 122.4, SE 2.2 mmHg) from the controls (Mean 123.2, SE 3.2 mmHg, *p* = 0.42). There were also no significant differences in the maximum exertional change in SBP or DBP during exercise (Figure 1). The wall sit maneuver was found to cause the largest change in systolic and diastolic pressure in both groups.

### ABPM data collection and protocol completion rates

The completion rate of the protocol by the TAD group (77%) was not significantly different from the control group (78%). There was no statistical difference in the mean age of subjects with errors (Mean 43.5, SD 14.7 years) and without errors (Mean 45.3, SD 14.2 years) or mean BMI of subjects with errors (Mean 28, SD 6.6 kg/m^2^) and without errors (Mean 27, SD 6.4 kg/m^2^). Mean individual exercise completion rates from highest to lowest were hand grip (100%), bicep curls (96%), wall sit (93%), stationary cycling (91%), and leg raise (85%). Review of error logs from the ABPM sentinel software showed that incomplete measurements were primarily caused by failure to obtain ABPM measurements after more than two attempts (60%) or inability to complete the exercise (40%). All incomplete cases were able to successfully complete at least three of the five exercises, and 80% completed at least four exercises.

### Safety of exercise protocol

The exercise protocol was not terminated due to severe exertional hypertension (SBP > 230 mmHg) or chest pain. Exercises that caused SBP to exceed 180 mmHg were: wall sits (7/28, 25%), bicep curls (2/29, 7%), stationary bicycling (1/31, 3%), and hand grips (2/31, 6%). Exercises that caused DBP to exceed 100 mmHg were: wall sits (19/28, 68%), bicep curls (8/29, 24%), stationary cycling (3/31, 10%), hand grips (6/31, 19%), and leg raises (7/16, 44%). The mean rate pressure product of stationary cyclists was 14213 (8555 to 25200).

### Weekly activity level and exercise BP

**Figure 2:**
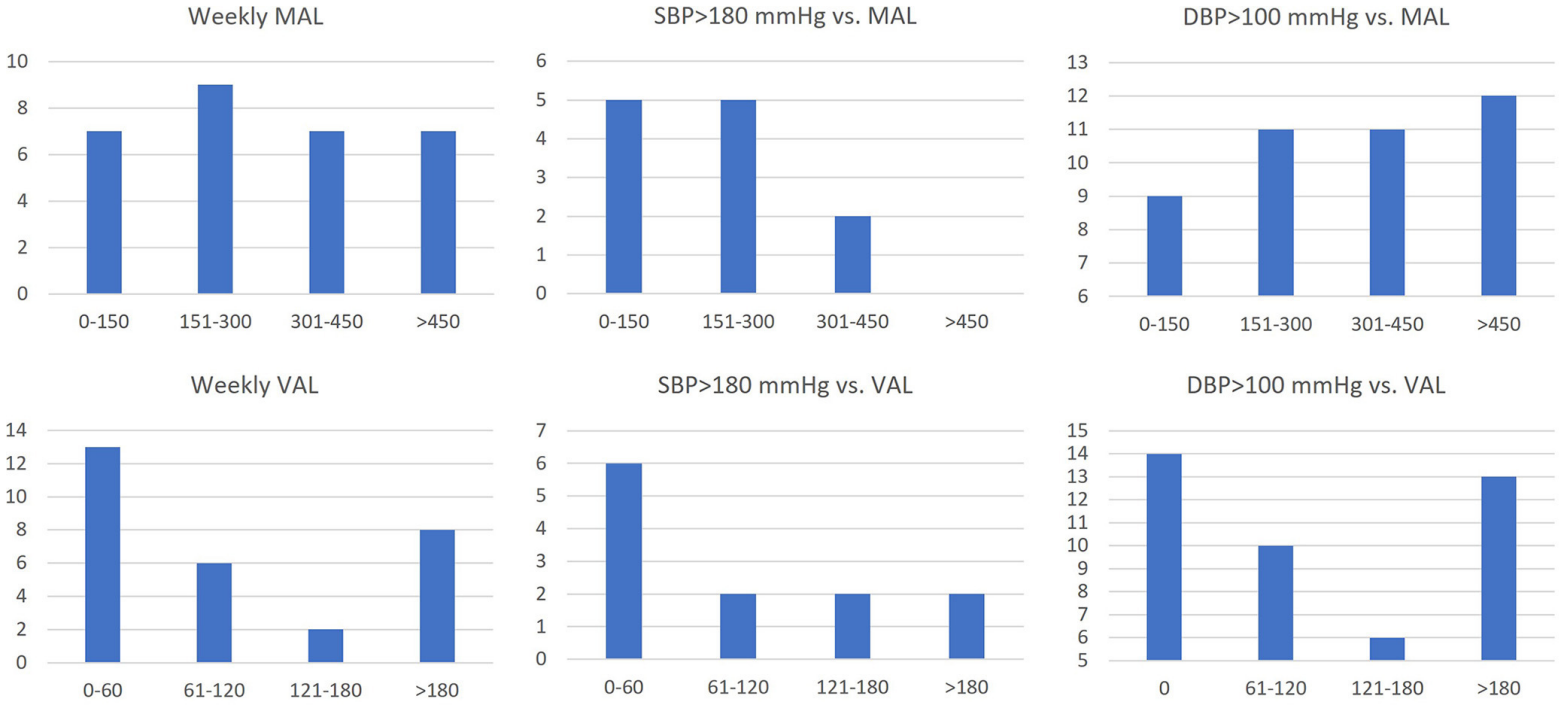
Number of exertional SBP measurements >180 mmHg (*n* = 12) or exertional DBP measurements>100 mmHg (*n* = 43) correlated with weekly moderate activity level (MAL) and vigorous activity level (VAL) in TAD patients. Y-axis: number of patients or exercise measurements; x-axis: weekly activity level (min/week).

Self-reported weekly activity levels were categorized as moderate (MAL) or vigorous (VAL) based on the WHO GPAQ criteria (Cleland et al., 2014). 74% of TAD patients reported at least 150 min MAL. The median MAL in TAD patients was 300 min/week (0–1,260 min). The median VAL in TAD patients was 120 min per week (0–600). Increased MAL corresponded with lower exercise SBP, with only 17% of exercise SBP > 180 mmHg occurring in TAD patients with MAL greater than the median. However, increased MAL did not correspond as strongly with lower exercise DBP, with 53% of exercise DBP > 100 mmHg occurring in TAD patients with MAL greater than the median. Increased VAL also corresponded modestly with lower exercise SBP, with 33% of exercise SBP > 180 mmHg occurring in TAD patients with VAL greater than the median. Increased VAL also corresponded weakly with exercise DBP, with 44% of exercise DBP > 100 mmHg occurring in TAD patients with VAL greater than the median. Figure 2 illustrates the association between minutes of self-reported weekly activities and exertional BP responses.

### Borg effort and BP

Borg effort scores were obtained during exercise for twelve TAD patients. Average Borg scale scores obtained for each exercise in TAD were leg raise (Mean 7, SE 0.6), hand grip (Mean 4, SE 0.5), stationary bicycling (Mean 4, SE 0.4), bicep curls (Mean 3, SE 0.5), and wall sit (Mean 5, SE 0.7).

Borg scores were separated into low (1–3), medium (4–6), and high (7–10) exertion groups for each exercise. Using one-tailed *t*-tests to examine for higher BP change with increased exertion, a significant difference in SBP was seen during wall sits between the low and high Borg groups (Mean 155.0, SE 5.0 mmHg vs. Mean 178.3, SE 8.8 mmHg, *p* = *0.0*4) as well as medium and high Borg groups (Mean 154.0, SE 4.3 mmHg vs. Mean 178.3, SE 8.8 mmHg, *p* = *0.0*3). No significant changes in SBP or DBP were seen among reported exertional groups in other exercises.

### BP response by aortic phenotype

TAD patients were separated by aortic phenotype into three subgroups (aneurysm, stent graft or repair, and dissection) for comparison. No significant BP changes were found when comparing cases with thoracic aortic aneurysm (*n* = 18) and those without (*n* = 9). No significant BP changes were found when comparing cases with aortic stent graft or repair (*n* = 8) and those without (*n* = 17).

Maximum DBPs were lower in participants with thoracic aortic dissection (*n* = 8) compared to other participants (*n* = 23) during hand grip (Mean 82.0, SE 3.2 mmHg vs. Mean 95.7, SE 2.4 mmHg, *p* = *0.0*01), cycle (Mean 76.5, SE 4.5 mmHg vs. Mean 86.9, SE 2.3 mmHg, *p* = *0.0*03), bicep curls (Mean 79.0, SE 3.9 mmHg vs. Mean 95.7, SE 2.4 mmHg, *p* = *0.0*01), and wall sit (Mean 93.5, SE 7.1 mmHg vs. Mean 107.6, SE 1.9 mmHg, *p* = 0*.0*48). Post-exercise DBP was also lower in participants with aortic dissection (Mean 67.3, SE 3.7 mmHg) than other TAD participants (Mean 80.3, SE 1.8 mmHg, *p* = 0.004). Maximum pressures did not change significantly between the first and second exercise circuits. Table 2 shows the demographic characteristics of dissection and non-dissection patients.

**Table 2:** Demographic characteristics of TAD cases stratified by history of aortic dissection.

	**Dissection (*****n** =* **8)**	**No Dissection (*****n** =* **23)**	***p*** **-value**
Age	46 (15)	46 (13)	0.97
Male/Female	5/3	17/6	n/a
BMI	28.4 (8.3)	27.1 (6.4)	0.71
Weekly MAL	71 (138)	223 (306)	0.07
Weekly VAL	71 (111)	134 (184)	0.27
Beta-blocker	6 (75%)	6 (26%)	n/a
Non-beta-blocker Antihypertensive	6 (75%)	10 (43%	n/a

*Data shown as mean (SD). BMI, body mass index (kg/m^2^); Weekly MAL, weekly moderate activity level (min/week); weekly VAL, weekly vigorous activity level (min/week); BB: beta-blocker; AH: anti-hypertensive. Medication usage shown as the percent taking any dose of any medication in category*.

### Medication use

Medication usage among the TAD group included: no medication (*n* = 12), beta blocker alone (*n* = 3), antihypertensive alone (*n* = 7), or both beta blocker and antihypertensive (*n* = 9). Among those taking medications, 58% reported taking one medication, 26% reported taking two medications, and 16% reported taking three or more medications. No significant difference in SBP or DBP was found between those using no medication and those using beta blockers alone, antihypertensives alone, or both beta blockers and antihypertensives. A significant difference in maximum exercise heart rate was seen between those taking beta blockers (Mean 92.8, SE 5.6 BPM) and those not taking beta blockers (Mean 112.0, SE 5.0 BPM, *p* = 0.02).

### Post-study survey

More than half of respondents (*n* = 10) expressed increased confidence in their ability to participate in exercise safely. Two-thirds of respondents said they valued the experience and would participate in the study again. Four respondents wrote that participation in exercise had improved their mental health.

## Discussion

Thoracic aortic aneurysms and dissections (TAD) are the 13th-leading cause of death in the United States with an estimated incidence of 3-4 cases per 100,000 persons per year (LeMaire and Russell, 2011). Previous studies have proposed strategies for TAD patients to maintain an active lifestyle (Chaddha et al., 2015; Spanos et al., 2018), but there are no evidence-based exercise guidelines for TAD or documented evidence about the safety of exercise for TAD patients. Anecdotal evidence that exercise can trigger acute aortic dissections has led to long-standing activity restrictions on TAD patients (Chaddha et al., 2015). To address these gaps, we developed a standardized and reproducible exercise protocol for systematic assessment of exercise capacity and blood pressure response in TAD patients and controls. In this study, we demonstrated the safety and feasibility of an exercise regimen in a high-risk cohort of TAD patients. There were no significant differences in the maximum exertional SBP, DBP, or change from baseline between the TAD and control groups. We observed that Borg effort scores reliably predict large changes in systolic and diastolic pressure and may be a useful proxy to minimize exertional hypertension. With appropriate guidance, we found that most TAD patients can safely engage in moderate intensity exercises, even after aortic dissection or surgery, regardless of medication use or disease status.

TAD patients are frequently counseled to minimize strenuous activities due to their perceived risk for sudden death due to an acute aortic rupture and are therefore hesitant to exercise. In follow up surveys, more than half of participants expressed increased confidence in exercising after they completed the study protocol. Several participants wrote that they experienced improved mental health and quality of life, which has been corroborated in other studies (Pasadyn et al., 2021). Regular exercise can also protect against exertional hypertension (Dimeo et al., 2012). Participants who reported more weekly activity minutes at baseline developed fewer hypertensive episodes during exercise. The psychological benefits of exercise, combined with individualized exercise prescriptions based on observational data, could motivate patients to undertake beneficial lifestyle changes.

There were several limitations to our study. Exercise ABPM presents technical challenges that may limit the accuracy of measurements because monitors are sensitive to movement or vibration. As specified in guidelines for blood pressure measurement, we ensured that the arm was immobilized while the cuff inflated by performing the measurements while the participant was seated or supine, and by bracing the measurement arm if needed (Muntner et al., 2019). For this reason, our protocol largely succeeded during moderate intensity exercises, but may not be adaptable to more vigorous activities or to other exercises. Although there were no statistical differences between the TAD and control groups, differences in the age and gender of the case and control groups may have confounded the results. No Borg scores for controls were obtained and therefore differences between perceived exertion in TAD and controls could not be investigated. This small single-center sample may not be representative of all TAD patients.

This study was not intended to collect longitudinal data on cardiovascular outcomes, which may be addressed in a future multicenter trial. We plan to expand the study protocol to include additional moderate intensity exercises and to investigate the effect of training on serial blood pressure responses to exercise.

## Data availability statement

The raw data supporting the conclusions of this article will be made available by the authors, without undue reservation.

## Ethics statement

The studies involving human participants were reviewed and approved by Committee for the Protection of Human Subjects at the University of Texas Health Science Center at Houston (UTHealth). The patients/participants provided their written informed consent to participate in this study.

## Author contributions

JL, AB, MH, and SP contributed to the study design and data acquisition. JL, AB, and SP analyzed and interpreted the data. JL, AB, JB, and SP drafted and revised the manuscript. All authors contributed toward the final approval of the manuscript.

## Conflict of interest

The authors declare that the research was conducted in the absence of any commercial or financial relationships that could be construed as a potential conflict of interest.

## Publisher's note

All claims expressed in this article are solely those of the authors and do not necessarily represent those of their affiliated organizations, or those of the publisher, the editors and the reviewers. Any product that may be evaluated in this article, or claim that may be made by its manufacturer, is not guaranteed or endorsed by the publisher.
